# A Multicriteria Decision Making Approach Based on Fuzzy Theory and Credibility Mechanism for Logistics Center Location Selection

**DOI:** 10.1155/2014/347619

**Published:** 2014-08-19

**Authors:** Bowen Wang, Haitao Xiong, Chengrui Jiang

**Affiliations:** ^1^School of Computer Science and Information Engineering, Beijing Technology and Business University, Beijing 100048, China; ^2^School of Computer Science and Engineering, Beihang University, Beijing 100191, China; ^3^Collaborative Innovation Centre for State-Owned Assets Administration, Beijing 100048, China

## Abstract

As a hot topic in supply chain management, fuzzy method has been widely used in logistics center location selection to improve the reliability and suitability of the logistics center location selection with respect to the impacts of both qualitative and quantitative factors. However, it does not consider the consistency and the historical assessments accuracy of experts in predecisions. So this paper proposes a multicriteria decision making model based on credibility of decision makers by introducing priority of consistency and historical assessments accuracy mechanism into fuzzy multicriteria decision making approach. In this way, only decision makers who pass the credibility check are qualified to perform the further assessment. Finally, a practical example is analyzed to illustrate how to use the model. The result shows that the fuzzy multicriteria decision making model based on credibility mechanism can improve the reliability and suitability of site selection for the logistics center.

## 1. Introduction

In modern society, logistics systems have become essential in ensuring economic development and the normal function of the society and the suitability of site selection for the logistics center have direct impact on the efficiency of logistics systems; therefore, a reasonable approach of site selection must be adopted. When selecting the location for the logistics center, not only quantitative factors like costs and distances but also qualitative factors such as natural conditions and infrastructure conditions should be taken into consideration; unlike traditional approaches' ignorance of qualitative factors, multicriteria decision making approach based on fuzzy theory takes both groups of factors into consideration. Hence, it is of great importance to conduct researches on multicriteria decision making approach based on fuzzy theory.

Fuzzy theory was firstly introduced to measure the impacts of qualitative factors on results in real life, which cannot be measured by traditional algorithms. An analytical network process tool was used to select suitable facility locations by Tuzkaya et al. [1]. The analytic hierarchy process (AHP) is a structured technique which has been widely used for organizing and analyzing complex decisions and can take both qualitative and quantitative factors into consideration. Badri offered a method which combined AHP and goal program modeling approach for international facility location problem [2]. Intelligent algorithm like genetic algorithm was also used in the multicriteria decision making problem. For example, a modified multicriterion optimization genetic algorithm was proposed for order distribution in collaborative supply chain [3]. A combination of genetic algorithm and AHP model was developed to solve distribution network problems in supply chain management [4]. But these methods mentioned above could only deal with facility location problem under certain environment. To solve distribution network problems under uncertain environment, new methods were proposed; for instance, an algorithm for facility site selection based on fuzzy theory and hierarchical structure analysis was proposed to deal with facility site selection problem under uncertain environment [5]. Chan et al. introduced a fuzzy-AHP approach in the global supplier selection problem [6]. In order to resolve the ambiguity of concepts that are associated with human being's judgments, a fuzzy TOPSIS model under group decision making to solve the facility location selection problem was built for evaluating facility locations [7, 8]. Liu et al. have introduced an approach which combined fuzzy theory and rough sets in the distribution center location problem [9]. Kahraman et al. used four fuzzy multiattribute group decision making approaches for alternative locations, with which uncertainty and vagueness from subjective perception can be effectively represented and reached to a more effective decision [10]. Chan and Prakash have proposed a new kind of maintenance policy selection approach using the fuzzy multicriteria decision making approach [11]. Lee and Lin introduced a fuzzy simple additive weighting approach with objective and subjective criteria under group decision making for location selection [12]. In order to calculate criteria values under uncertain environment to evaluate and select the suitable location for building logistic center, a fuzzy TOPSIS model was built by Zadeh [13]. However, the literature listed above neglects the impacts of consistency and historical assessment accuracy of decision makers on the results and thus cannot ensure the accuracy of site selection result.

In response to the problem above, this paper firstly defined the criteria set of the logistics center location selection in Section 2 and then introduced the definitions and notations related to the fuzzy theory and the credibility mechanism, including the linguistic variables, the membership function, the consistency ranking mechanism, the spearman ranking correlation coefficient, and the historical assessment accuracy in Section 3. In Section 4, the multicriteria decision making model based on fuzzy theory and credibility mechanism is established. In the end, a numerical example is analyzed to illustrate how to use the model. The result shows that the approach proposed in this paper can improve the reliability of the result for site selection.

## 2. Defining and Selecting Location Criteria

Since the criteria will be used to evaluate the potential location for the logistics center, it is of great importance to select a justified set of criteria to ensure the rationality of final result [14]. In this paper, factors shown in Table 1 will be used as the criteria set for the logistics center location selection.

(*1) Cost.* In the process of location selection, factors such as land cost, operation cost, and labor costs should be taken into comprehensive consideration.

(*2) Distance to Supplier*. It is also called internal traffic convenience, having great effect on the transportation cost.

(*3) Distance to Customer*. It is also called external traffic convenience, affecting the distribution efficiency and the transportation cost directly.

(*4) Natural Conditions.* They mainly include the meteorological conditions, the terrain conditions, and the hydrologic conditions.

(*5) Conformance to Other Means of Transportation*. In modern logistics systems, intermodal transportation is usually adopted; therefore, good compatibility on different mode of transportation will ensure the efficiency of the transportation of cargos.

(*6) Infrastructure Conditions*. They mainly include the reliability of the infrastructure and convenience of communication systems.

## 3. Definitions and Notations

### 3.1. Defining the Linguistic Variables

In this paper, linguistic variables ranging from a scale of 1 to 9 for rating the criteria and the potential locations for the logistics center will be used, as illustrated in Tables 2 and 3.

### 3.2. Defining the Membership Function

**Figure 1:**
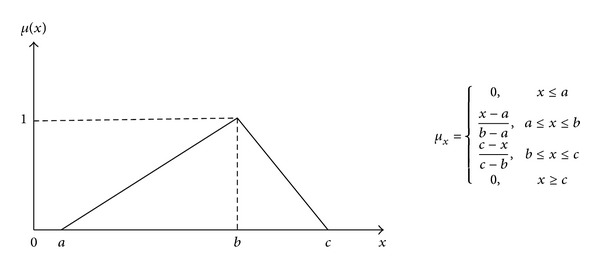
Triangular membership function.

Triangular membership function, trapezoidal membership function, Gaussian membership function, and bell-shaped membership function are commonly used [15]; in this paper, the triangular membership function will be used and the membership function of triangular fuzzy number *x* is defined as illustrated in Figure 1.

### 3.3. Defining the Credibility Mechanism

The credibility mechanism proposed in this paper mainly includes two aspects: the consistency level and the historical assessment accuracy; they are introduced as follows.

#### 3.3.1. Defining the Consistency Ranking Mechanism

*C*_*i*_ and  *C*_*j*_ are two random criterias in the criterion set; *U*_*ij*_ is the qualitative value used to describe the significance of criteria *C*_*i*_ and *C*_*j*_.* Stipulation*. If an expert finds that *C*_*i*_ is more important than *C*_*j*_, then *U*_*ij*_ = 1, *U*_*ji*_ = 0; if *C*_*i*_ is as important as *C*_*j*_, then *U*_*ij*_ = *U*_*ji*_ = 0.5; if *C*_*j*_ is more important than *C*_*i*_, then *U*_*ij*_ = 0, *U*_*ji*_ = 1. So, a binary qualitative ranking matrix can be conducted as *U* = (*U*_*ij*_)_*m*×*n*_, *U*_*ij*_ can only be 0,0.5 or 1, and *S*_*i*_ is the sum of elements in row *i*. The value of *U*_*ij*_ also satisfies the following equations:

(1) $$\begin{array}{c} U_{ij}+U_{ji}=1;\;\left(i\neq j;i=1,2,\ldots ,m;j=1,2,\ldots ,m\right),\\ U_{ij}=U_{ji}=0.5;\;\left(i=j;i=1,2,\ldots ,m;j=1,2,\ldots ,m\right). \end{array}$$

Then, according to the ranking of numeric value of *S*_*i*_, the consistency ranking of the significance about the criterion set provided by that expert can be decided.

#### 3.3.2. Defining the Spearman Ranking Correlation Coefficient

Spearman ranking correlation coefficient is used to solve problems which are hard to calculate in numbers or to analyze by using simple correlation coefficient [16, 17]. In this paper, we can use spearman ranking correlation coefficient to conduct consistency check on criterion rankings provided by different experts.

Define the ranking result provided by expert *p* as *A*_*p*_ = (*a*_1*p*_, *a*_2*p*_,…, *a*_*ip*_,…, *a*_*mp*_), in which *a*_*ip*_ is the *i*th element in the ranking result provided by expert *p*.

Define the ranking result provided by expert *q* as *A*_*q*_ = (*a*_1*q*_, *a*_2*q*_,…, *a*_*iq*_,…, *a*_*mq*_), in which *a*_*iq*_ is the *i*th element in the ranking result provided by expert *q*.

Then the ranking correlation coefficient of rankings provided by expert *p* and expert *q*: the spearman ranking correlation coefficient is *ρ*_*pq*_.

Defining $\bar{\rho_{p}}$ as the mean value of the spearman ranking correlation coefficient, it represents the consistency level of a certain expert.

#### 3.3.3. Defining the Historical Assessment Accuracy

The accuracy of the historical assessment has great effect on the credibility of a certain expert; therefore, in this section, define *N*_*t*_(*p*, *i*) as the number of times expert *p* provided right performance rating for criterion *i* in the historical assessment; define *N*_*f*_(*p*, *i*) as the number of times expert *p* provided wrong performance rating for criterion *i* in the historical assessment. *A*_*pi*_ is the historical assessment accuracy of expert *p* with respect to criterion *i*; it is based on the mutual history of expert *p* and criterion *i*. $\bar{A_{p}}$ is the mean value of the historical assessment accuracy of expert *p* with respect to all the criteria; it represents the accuracy level of a certain expert.

#### 3.3.4. Define the Credibility of the Experts

From the analysis above, the credibility of an expert is defined as the comprehensive effect of the consistency level and the accuracy level of an expert. Therefor, the credibility of expert *p* could be defined as

(2) $$\begin{array}{c} C_{p}=\alpha \bar{\rho_{p}}+\left(1-\alpha \right)\bar{A_{p}}. \end{array}$$

The main function of the credibility of the experts is to eliminate the rankings failing to pass the credibility check. The standard of elimination calculates the value of the credibility of a certain expert; experts can continue to participate in the following assessment only when the value exceeds certain threshold value *T*; otherwise the assessment result of the expert will be eliminated.

## 4. Fuzzy Multicriteria Decision Making Model Establishing

Suppose that there is a committee of *n* experts (*k*_1_, *k*_2_,…, *k*_*n*_) who are responsible for assessing the performance of *m* potential locations *L* = (*L*_1_, *L*_2_,…, *L*_*m*_) in respect to *k* criteria *C* = (*C*_1_, *C*_2_,…, *C*_*k*_), the weights of the criteria are denoted by *W*_*i*_  (*i* = 1,2,…, *m*); the performance ratings of expert *k* for location *j* with respect to criterion *i* are denoted by *X*_*ijk*_  (*i* = 1,2,…, *k*; *j* = 1,2,…, *m*; *k* = 1,2,…, *n*); *U* ^*n*^ = (*U*_*ij*_)_*m*×*n*_ is the binary qualitative ranking matrix of a certain logistic center provided by expert *k*_*n*_, and *U*_*ij*_ is the qualitative value used to describe the significance of criteria *C*_*i*_ and *C*_*j*_. The multicriteria decision making model based on fuzzy theory and consistency mechanism can be described by the following steps.

*Step 1*. Conduct expert selection according to the credibility mechanism.

*Step 1.1.* Define *S*_*i*_ as the sum of elements in row in *U*_*ij*_; compute *S*_*i*_:

(3) $$\begin{array}{c} S_{i}=\overset{m}{\underset{k=1}{\sum }}U_{ik};\;\left(i=1,2,\ldots ,m\right). \end{array}$$

*Step 1.2*. Get the consistency ranking for the 6 criteria.

*Step 1.3.* Compute the spearman ranking correlation coefficient *ρ*_*pq*_:

(4) $$\begin{array}{c} \rho_{pq}=1-\frac{\left[6\sum_{i=1}^{m}{\left(a_{ip}-a_{iq}\right)}^{2}\right]}{m\left(m^{2}-1\right)}. \end{array}$$

*Step 1.4.* Compute the mean value of the spearman ranking correlation coefficient.

In order to take the ranking correlation coefficient of the experts into a comprehensive comparison and select the appropriate experts for further assessment, compute the mean value of the spearman ranking correlation coefficient $\bar{\rho_{p}}$:

(5) $$\begin{array}{c} \bar{\rho_{p}}=\frac{\sum_{q=1}^{k}\rho_{pq}}{k}. \end{array}$$

*Step 1.5.* Compute the historical assessment accuracy of expert *p* with respect to criterion *i*:

(6) $$\begin{array}{c} A_{pi}=\frac{N_{t}\left(p,i\right)}{N_{t}\left(p,i\right)+N_{f}\left(p,i\right)};\;0\leq A_{pi}\leq 1. \end{array}$$

*Step 1.6.* Compute the mean value of the historical assessment accuracy of the experts:

(7) $$\begin{array}{c} \bar{A_{p}}=\frac{\sum_{i=1}^{k}A_{pi}}{p}. \end{array}$$

*Step 1.7* (*compute the credibility of the experts according to (1)*). Experts can continue to participate in the following assessment only when the value of the credibility exceeds certain threshold value *T*; otherwise the assessment result of the expert will be eliminated.

*Step 2* (*compute the aggregate fuzzy weights of each criteria*). The aggregate fuzzy rating for criterion *i* of all the experts is described as *R*_*i*_ = (*a*_*i*_, *b*_*i*_, *c*_*i*_), with

(8) $$\begin{array}{c} R_{i}=\left(a_{i},b_{i},c_{i}\right);\\ a_{i}=\min\left\{a_{ik}\right\};\;b_{i}=\frac{1}{k}\overset{k}{\underset{k=1}{\sum }}\left\{b_{ik}\right\};\;c_{i}=\max\left\{c_{ik}\right\}. \end{array}$$

*Step 3* (*evaluate the aggregate fuzzy rating of potential locations*). The aggregate fuzzy rating for potential location *j* with respect to criterion *i* of all the experts is described as *R*_*ij*_ = (*a*_*ij*_, *b*_*ij*_, *c*_*ij*_), with

(9) $$\begin{array}{c} R_{ij}=\left(a_{ij},b_{ij},c_{ij}\right);\\ a_{ij}=\min\left\{a_{ijk}\right\};\;b_{ij}=\frac{1}{k}\overset{k}{\underset{k=1}{\sum }}\left\{b_{ijk}\right\};\;c_{ij}=\max\left\{c_{ijk}\right\}. \end{array}$$

*Step 4* (*normalize the aggregate fuzzy ratings*). In order to bring the various criteria scales into a comparable one, the aggregate fuzzy ratings must be normalized; define $\bar{R_{ij}}$ as the fuzzy rating for *R*_*ij*_ after being normalized, with

(10) Rij¯=(aijcij,bijcij,cijcij), fi∈B,B:criteria  having  positive  effects  on⁡  location  selection,Rij¯=(aijcij,aijbij,aijaij), fi∈C,C:criteria  having  negative  effects  on⁡  location  selection.

*Step 5* (*calculate the final fuzzy evaluation for potential locations*). Considering the different importance of each criterion, define the final fuzzy evaluation value of each potential location as *p*_*i*_, with

(11) $$\begin{array}{c} p_{i}=\overset{n}{\underset{j=1}{\sum }}\bar{R_{ij}}\times R_{i},\;i=1,2,\ldots ,m. \end{array}$$

*Step 6* (*compare the potential locations*). *p*_*i*_ is triangular fuzzy number, so it is very difficult for experts to determine which location is the best directly. This paper conducts a pairwise comparison of *p*_*i*_ and *p*_*j*_ to show what is the preferable over two potential locations *L*_*i*_ and *L*_*j*_.

*Step 6.1*. Define the difference between *L*_*i*_ and *L*_*j*_ as

(12) $$\begin{array}{c} Z_{ij}=p_{i}-p_{j}. \end{array}$$

*Step 6.2* (*define the fuzzy preference matrix*). *E* = [*e*_*ij*_] is the fuzzy preference matrix, *e*_*ij*_ represents the degree of preference of location *L*_*i*_ over location *L*_*j*_  (*i*, *j* = 1,2,…, *m*), and the fuzzy preference relation between *L*_*i*_ and *L*_*j*_ is defined as

(13) $$\begin{array}{c} e_{ij}=\frac{S_{1}}{S},\;S>0,\;\;\text{with}\;S_{1}=\int_{x>0}u_{z_{ij}}\left(x\right)dx,\\ s=\int_{x>0}u_{z_{ij}}\left(x\right)dx+\int_{x<0}u_{z_{ij}}\left(x\right)dx. \end{array}$$

*Step 6.3.* Define the preference relation between *L*_*i*_ and *L*_*j*_ as

(14) $$\begin{array}{c} L_{i}>L_{j},\;e_{ij}>0.5,\\ L_{i}=L_{j},\;e_{ij}=0.5,\\ L_{i}<L_{j},\;e_{ij}<0.5. \end{array}$$

## 5. Application

A new logistics center is needed in a city. There are three potential locations, *L*_1_, *L*_2_, *L*_3_, and five experts, *K*_1_, *K*_2_, *K*_3_, *K*_4_, *K*_5_, intended to select the best location in respect to 6 criteria shown in Table 1. The criteria are compared in pairs by the experts, and the binary qualitative ranking matrix provided by the 5 experts is shown as follows:

(15) $$\begin{array}{c} U^{1}=\left[\begin{array}{cccccc} 0.5 & 1 & 1 & 1 & 1 & 1\\ 0 & 0.5 & 1 & 1 & 1 & 1\\ 0 & 0 & 0.5 & 1 & 1 & 1\\ 0 & 0 & 0 & 0.5 & 1 & 1\\ 0 & 0 & 0 & 0 & 0.5 & 1\\ 0 & 0 & 0 & 0 & 0 & 0.5 \end{array}\right],\\ U^{2}=\left[\begin{array}{cccccc} 0.5 & 1 & 1 & 1 & 1 & 1\\ 0 & 0.5 & 1 & 1 & 1 & 1\\ 0 & 0 & 0.5 & 1 & 1 & 1\\ 0 & 0 & 0 & 0.5 & 1 & 0\\ 0 & 0 & 0 & 0 & 0.5 & 0\\ 0 & 0 & 0 & 1 & 1 & 0.5 \end{array}\right],\\ U^{3}=\left[\begin{array}{cccccc} 0.5 & 1 & 1 & 1 & 1 & 1\\ 0 & 0.5 & 1 & 1 & 1 & 1\\ 0 & 0 & 0.5 & 0 & 1 & 1\\ 0 & 0 & 1 & 0.5 & 1 & 1\\ 0 & 0 & 0 & 0 & 0.5 & 1\\ 0 & 0 & 0 & 0 & 0 & 0.5 \end{array}\right],\\ U^{4}=\left[\begin{array}{cccccc} 0.5 & 1 & 1 & 1 & 1 & 1\\ 0 & 0.5 & 0 & 1 & 1 & 1\\ 0 & 1 & 0.5 & 1 & 1 & 1\\ 0 & 0 & 0 & 0.5 & 0 & 1\\ 0 & 0 & 0 & 1 & 0.5 & 1\\ 0 & 0 & 0 & 0 & 0 & 0.5 \end{array}\right],\\ U^{5}=\left[\begin{array}{cccccc} 0.5 & 1 & 0 & 1 & 1 & 1\\ 0 & 0.5 & 0 & 1 & 1 & 1\\ 1 & 1 & 0.5 & 1 & 1 & 1\\ 0 & 0 & 0 & 0.5 & 1 & 0\\ 0 & 0 & 0 & 0 & 0.5 & 0\\ 0 & 0 & 0 & 1 & 1 & 0.5 \end{array}\right]. \end{array}$$

The steps to be taken are described as follows.

*Step 1.* Conduct expert selection according to the credibility mechanism.

*Step 1.1.* Compute the sum of the elements of rows in *U* ^1^ using (3):

(16) $$\begin{array}{c} S_{1}^{1}=5.5;\;\;S_{2}^{1}=3.5;\;\;S_{3}^{1}=3.5;\\ S_{4}^{1}=2.5;\;\;S_{5}^{1}=1.5;\;\;S_{6}^{1}=0.5. \end{array}$$

*Step 1.2.* Get the consistency ranking for the 6 criteria.

 The consistency ranking for the 6 criteria provided by expert *k*_1_ is *C*_1_ > *C*_2_ > *C*_3_ > *C*_4_ > *C*_5_ > *C*_6_.
 The consistency ranking for the 6 criteria provided by expert *k*_2_ is *C*_1_ > *C*_2_ > *C*_3_ > *C*_6_ > *C*_4_ > *C*_5_.
 The consistency ranking for the 6 criteria provided by expert *k*_3_ is *C*_1_ > *C*_2_ > *C*_4_ > *C*_3_ > *C*_5_ > *C*_6_.
 The consistency ranking for the 6 criteria provided by expert *k*_4_ is *C*_1_ > *C*_3_ > *C*_2_ > *C*_5_ > *C*_4_ > *C*_6_.
 The consistency ranking for the 6 criteria provided by expert *k*_5_ is *C*_3_ > *C*_1_ > *C*_2_ > *C*_6_ > *C*_4_ > *C*_5_.

*Step 1.3.* Compute the spearman ranking correlation coefficient using (4).

 The spearman ranking correlation coefficient between *k*_1_ and *k*_2_ is *ρ*_12_ = 0.83.
 The spearman ranking correlation coefficient between *k*_1_ and *k*_3_ is *ρ*_13_ = 0.94.
 The spearman ranking correlation coefficient between *k*_1_ and *k*_4_ is *ρ*_14_ = 0.89.
 The spearman ranking correlation coefficient between *k*_1_ and *k*_5_ is *ρ*_15_ = 0.66.

*Step 1.4.* Compute the mean value of the spearman ranking correlation coefficient using (5).

The mean value of the spearman ranking correlation coefficient between expert *k*_1_ and all the other experts is

(17) ρ1¯=ρ12+ρ13+ρ14+ρ154=0.83+0.94+0.89+0.664=0.83.

The mean value of the spearman ranking correlation coefficient between expert *k*_2_ and all the other experts is

(18) ρ2¯=ρ21+ρ23+ρ24+ρ254=0.83+0.66+0.89+0.834=0.74.

The mean value of the spearman ranking correlation coefficient between expert *k*_3_ and all the other experts is

(19) ρ3¯=ρ31+ρ32+ρ34+ρ354=0.94+0.66+0.71+0.434=0.69.

The mean value of the spearman ranking correlation coefficient between expert *k*_4_ and all the other experts is

(20) ρ4¯=ρ41+ρ42+ρ43+ρ454=0.89+0.89+0.71+0.714=0.8.

The mean value of the spearman ranking correlation coefficient between expert *k*_5_ and all the other experts is

(21) ρ5¯=ρ51+ρ52+ρ53+ρ544=0.66+0.83+0.43+0.714=0.66.

*Step 1.5* (*compute the historical assessment accuracy of all the experts*). The mutual history of the experts with respect to each criterion is shown in Table 4.

Calculate the historical assessment accuracy of the experts with respect to each criterion using (6); see Table 5.

*Step 1.6.* Compute the mean value of the historical assessment accuracy of the experts using (7):

(22) A1¯=A11+A12+A13+A14+A15+A166=0.5+0.8+0.6+1+0.8+0.56=0.7,A2¯=A21+A22+A23+A24+A25+A266=0.57+1+1+0.5+0.6+16=0.78,A3¯=A31+A32+A33+A34+A35+A366=0.5+0.44+0.75+0.25+0.5+0.56=0.49,A4¯=A41+A42+A43+A44+A45+A466=1+0.4+1+0.67+1+16=0.85,A5¯=A51+A52+A53+A54+A55+A566=0.5+0.4+0.67+1+0.67+0.536=0.63.

*Step 1.7* (*compute the credibility of the experts using (2)*). In this paper, consider that the consistency level is as important as the accuracy level; set the weight *α* = 0.5:

(23) $$\begin{array}{c} C_{1}=0.5\bar{\rho_{1}}+0.5\bar{A_{1}}=0.77,\\ C_{2}=0.5\bar{\rho_{2}}+0.5\bar{A_{2}}=0.76,\\ C_{3}=0.5\bar{\rho_{3}}+0.5\bar{A_{3}}=0.69,\\ C_{4}=0.5\bar{\rho_{4}}+0.5\bar{A_{4}}=0.83,\\ C_{5}=0.5\bar{\rho_{5}}+0.5\bar{A_{5}}=0.65. \end{array}$$

In this paper, we set the credibility threshold value of the experts *T* = 0.7. According to the result above, experts *k*_1_, *k*_2_, and *k*_4_ pass the credibility check and perform the following assessments. The performance rating of the 6 criteria and the potential locations are shown in Tables 6 and 7.

*Step 2.* Compute the aggregate fuzzy weights of each criterion using (8); see Table 8.

*Step 3.* Evaluate the aggregate fuzzy rating of potential locations with respect to each criterion using (9); see Table 9.

*Step 4.* Normalize the aggregate fuzzy ratings using (10); see Table 10.

*Step 5.* Compute final evaluation for potential locations using (11); see Table 11.

The final fuzzy evaluation values are

(24) $$\begin{array}{c} p_{1}=\left(13.76,28.19,52\right),\\ p_{2}=\left(11,26.68,54\right),\\ p_{3}=\left(12.69,27.33,52\right). \end{array}$$

*Step 6.* Compare the potential locations using (12) and (13); results are as follows.

The fuzzy preference matrix is

(25) $$\begin{array}{c} E=\left(\begin{array}{ccc} 0.50 & 0.75 & 1\\ 0.25 & 0.50 & 0.41\\ 0 & 0.59 & 0.50 \end{array}\right). \end{array}$$

According to the computing result, compare the three potential locations using (14), *p*_1_ > *p*_3_ and *p*_3_ > *p*_2_; therefore, *p*_1_ is the best location to establish the logistics center.

## 6. Conclusions

The multiple-criteria decision making approach based on fuzzy theory and credibility mechanism proposed in this paper has taken both quantitative criteria and qualitative criteria under uncertain environment into consideration. Moreover, the credibility mechanism which is composed by the consistency level and the historical assessment accuracy solved the inaccuracy caused by the different credibility of experts and improved the reliability of the logistic center location selection. The method proposed in this paper is a better one in logistic center location than traditional algorithms.

## Figures and Tables

**Table 1:** Criteria for location selection.

Criterion	Unit of Measurement
Costs (*C*_1_) [10]	Quantitative
Distance to Suppliers (*C*_2_)	Quantitative
Distance to Customers (*C*_3_)	Quantitative
Natural Conditions (*C*_4_)	Qualitative
Conformance to other means of transportation (*C*_5_)	Qualitative
Infrastructure conditions (*C*_6_)	Qualitative

**Table 2:** Linguistic terms for objective ratings.

Linguistic Term	Membership function
Very poor (VP)	(1, 1, 3)
Poor (P)	(1, 3, 5)
Fair (F)	(3, 5, 7)
Good (G)	(5, 7, 9)
Very good (VG)	(7, 9, 9)

**Table 3:** Linguistic terms for criteria ratings.

Linguistic term	Membership function
Very low (VL)	(1, 1, 3)
Low (L)	(1, 3, 5)
Medium (M)	(3, 5, 7)
High (H)	(5, 7, 9)
Very high (VH)	(7, 9, 9)

**Table 4:** Mutual history of the experts with respect to each criterion.

Criterion	Experts									
	*k*_1_		*k*_2_		*k*_3_		*k*_4_		*k*_5_	
	*N*_*t*_(*p*, *i*)	*N*_*f*_(*p*, *i*)	*N*_*t*_(*p*, *i*)	*N*_*f*_(*p*, *i*)	*N*_*t*_(*p*, *i*)	*N*_*f*_(*p*, *i*)	*N*_*t*_(*p*, *i*)	*N*_*f*_(*p*, *i*)	*N*_*t*_(*p*, *i*)	*N*_*f*_(*p*, *i*)
*C*_1_	3	3	4	3	2	2	5	0	4	4
*C*_2_	4	1	3	0	4	5	2	3	2	3
*C*_3_	3	2	6	0	3	1	4	0	2	1
*C*_4_	4	0	2	2	2	6	6	3	5	0
*C*_5_	4	1	3	2	3	3	9	0	8	4
*C*_6_	2	2	6	0	3	3	8	0	9	8

**Table 5:** Credibility of experts with respect to each criterion.

Criterion	Experts				
	*k*_1_	*k*_2_	*k*_3_	*k*_4_	*k*_5_
*C*_1_	0.5	0.57	0.5	1	0.5
*C*_2_	0.8	1	0.44	0.4	0.4
*C*_3_	0.6	1	0.75	1	0.67
*C*_4_	1	0.5	0.25	0.67	1
*C*_5_	0.8	0.6	0.5	1	0.67
*C*_6_	0.5	1	0.5	1	0.53

**Table 6:** The performance rating of the 6 criteria.

Criterion	Experts assessments		
	*k*_1_	*k*_2_	*k*_4_
*C*_1_	VH	VH	VH
*C*_2_	M	L	M
*C*_3_	H	VH	VH
*C*_4_	VH	H	VH
*C*_5_	H	M	H
*C*_6_	VH	VH	H

**Table 7:** The performance rating of the potential locations.

Criterion	Potential locations	Experts assessments		
		*k*_1_	*k*_2_	*k*_4_
*C*_1_	*L*_1_	G	VG	G
	*L*_2_	G	G	VG
	*L*_3_	G	G	G

*C*_2_	*L*_1_	G	VG	VG
	*L*_2_	F	G	G
	*L*_3_	P	F	P

*C*_3_	*L*_1_	F	G	G
	*L*_2_	F	F	F
	*L*_3_	F	F	G

*C*_4_	*L*_1_	G	G	G
	*L*_2_	F	G	F
	*L*_3_	F	G	G

*C*_5_	*L*_1_	G	G	G
	*L*_2_	G	G	F
	*L*_3_	F	G	P

*C*_6_	*L*_1_	F	F	F
	*L*_2_	F	P	P
	*L*_3_	P	VP	VP

**Table 8:** The aggregate fuzzy weights of each criterion.

Criterion	Experts assessments			
	*k*_1_	*k*_2_	*k*_3_	Aggregate fuzzy weighs
*C*_1_	(7, 9, 9)	(7, 9, 9)	(7, 9, 9)	(7, 9, 9)
*C*_2_	(3, 5, 7)	(1, 3, 5)	(3, 5, 7)	(1, 4.33, 7)
*C*_3_	(5, 7, 9)	(7, 9, 9)	(7, 9, 9)	(5, 8.33, 9)
*C*_4_	(7, 9, 9)	(5, 7, 9)	(7, 9, 9)	(5, 8.33, 9)
*C*_5_	(5, 7, 9)	(3, 5, 7)	(5, 7, 9)	(3, 6.33, 9)
*C*_6_	(7, 9, 9)	(7, 9, 9)	(5, 7, 9)	(5, 8.33, 9)

**Table 9:** The aggregate fuzzy rating of potential locations with respect to each criterion.

Criterion	Potential locations	Experts assessments			
		*k*_1_	*k*_2_	*k*_3_	aggregate fuzzy ratings
*C*_1_	*L*_1_	(5, 7, 9)	(7, 9, 9)	(5, 7, 9)	(5, 7.66, 9)
	*L*_2_	(5, 7, 9)	(5, 7, 9)	(7, 9, 9)	(5, 7.66, 9)
	*L*_3_	(5, 7, 9)	(5, 7, 9)	(5, 7, 9)	(5, 7, 9)

*C*_2_	*L*_1_	(5, 7, 9)	(7, 9, 9)	(7, 9, 9)	(5, 8.33, 9)
	*L*_2_	(3, 5, 7)	(5, 7, 9)	(5, 7, 9)	(3, 6.33, 9)
	*L*_3_	(1, 3, 5)	(3, 5, 7)	(1, 3, 5)	(1, 3.66, 7)

*C*_3_	*L*_1_	(3, 5, 7)	(5, 7, 9)	(5, 7, 9)	(3, 6.33, 9)
	*L*_2_	(3, 5, 7)	(3, 5, 7)	(3, 5, 7)	(3, 5, 7)
	*L*_3_	(3, 5, 7)	(3, 5, 7)	(5, 7, 9)	(3, 5.66, 9)

*C*_4_	*L*_1_	(5, 7, 9)	(5, 7, 9)	(5, 7, 9)	(5, 7, 9)
	*L*_2_	(3, 5, 7)	(5, 7, 9)	(3, 5, 7)	(3, 5.66, 9)
	*L*_3_	(3, 5, 7)	(5, 7, 9)	(5, 7, 9)	(3, 6.33, 9)

*C*_5_	*L*_1_	(5, 7, 9)	(5, 7, 9)	(5, 7, 9)	(5, 7, 9)
	*L*_2_	(5, 7, 9)	(5, 7, 9)	(3, 5, 7)	(3, 6.33, 9)
	*L*_3_	(3, 5, 7)	(5, 7, 9)	(1, 3, 5)	(1, 5, 9)

*C*_6_	*L*_1_	(3, 5, 7)	(3, 5, 7)	(3, 5, 7)	(3, 5, 7)
	*L*_2_	(3, 5, 7)	(1, 3, 5)	(1, 3, 5)	(1, 3.66, 7)
	*L*_3_	(1, 3, 5)	(1, 1, 3)	(1, 1, 3)	(1, 1.66, 5)

**Table 10:** The final fuzzy evaluation value for potential locations.

Criterion	Normalized ratings		
	L_1_	L_2_	L_3_
*C*_1_	(0.56, 0.65, 1)	(0.56, 0.65, 1)	(0.56, 0.71, 1)
*C*_2_	(0.56, 0.60, 1)	(0.33, 0.47, 1)	(0.14, 0.27, 1)
*C*_3_	(0.33, 0.47, 1)	(0.43, 0.60, 1)	(0.33, 0.53, 1)
*C*_4_	(0.56, 0.78, 1)	(0.33, 0.63, 1)	(0.33, 0.70, 1)
*C*_5_	(0.56, 0.78, 1)	(0.33, 0.70, 1)	(0.11, 0.56, 1)
*C*_6_	(0.43, 0.60, 1)	(0.14, 0.27, 1)	(0.20, 0.60, 1)
*p*_*i*_	(0.56, 0.65, 1)	(0.56, 0.65, 1)	(0.56, 0.71, 1)

**Table 11:** The final fuzzy evaluation value for potential locations.

Criterion	Experts assessments		
	L_1_	L_2_	L_3_
*C*_1_	(3.92, 5.85, 9)	(3.92, 5.85, 9)	(3.92, 6.39, 9)
*C*_2_	(0.56, 3.00, 7)	(1.65, 3.9, 9)	(2.14, 3.17, 7)
*C*_3_	(1.65, 3.9, 9)	(2.15, 5, 9)	(1.65, 4.4, 9)
*C*_4_	(2.8, 5.50, 9)	(1.65, 5.25, 9)	(1.65, 5.83, 9)
*C*_5_	(2.68, 4.94, 9)	(1, 4.43, 9)	(2.33, 3.54, 9)
*C*_6_	(2.15, 5, 9)	(0.7, 2.25, 9)	(1, 5, 9)
*p*_*i*_	(13.76, 28.19, 52)	(11, 26.68, 54)	(12.69, 27.33, 52)
